# Mercury as a Geophysical Tracer Gas - Emissions from the Emperor Qin Tomb in Xi´an Studied by Laser Radar

**DOI:** 10.1038/s41598-020-67305-x

**Published:** 2020-06-26

**Authors:** Guangyu Zhao, Weixing Zhang, Zheng Duan, Ming Lian, Ningbin Hou, Yiyun Li, Shiming Zhu, Sune Svanberg

**Affiliations:** 1 Center for Optical and Electromagnetic Research, South China Academy of Advanced Optoelectronics, South China Normal University, University City Campus, Guangzhou, 510006 China; 2 Emperor Qin Shihuang’s Mausoleum Site Museum, Xi´an, 710600 China; 3 Department of Physics, Lund University, SE 221 00 Lund, Sweden

**Keywords:** Environmental sciences, Optics and photonics

## Abstract

Mercury is, because of its high vapor pressure and its prevalence in the atmosphere as atoms, an interesting geophysical tracer gas, also with potential archaeological applications. According to historical records dating back 2200 years, the mausoleum chamber of the “Terracotta Army Emperor” Qin in Xi´an, China, contains large amounts of liquid mercury, considered as an elixir of life at the time. We here report on measurements of the atmospheric contents of atomic mercury above the tomb mound performed with a mobile differential absorption lidar (light detection and ranging) system. Our measurements, which were performed from three different locations around the mound, indeed indicate elevated atmospheric mercury levels, with localizations, which correlate with previous *in situ* soil sampling results. Concentrations up to 27 ng/m^3^ were observed, significantly higher than the typical general pollutant level in the area which was found to be around 5–10 ng/m^3^. An out-flux of about 5×10^−8^ kg/s was estimated. Highly volatile mercury may be escaping through cracks, which developed in the structure over time, and our investigation supports ancient chronicle records on the tomb, which is believed never to have been opened/looted. Our findings also have bearings on the proposed use of mercury as a tracer gas for valuable ores and geothermal resource exploration, and also bring problematics around reliable nuclear waste long-term underground storage to mind.

## Introduction

The high vapor pressure of mercury also at ambient temperatures, makes it an interesting geophysical tracer gas. The fact that mercury is the only non-inert gas element, which is present in the atmosphere in atomic form makes optical spectroscopy methods particularly sensitive, and allows mercury detection down to the Atlantic background level of 1–2 ng/m^3^ (see, e.g. ref. ^1^ and references therein). Mercury is a highly toxic heavy metal, leading to an internationally suggested ban of its use, as implemented by many countries^2^. A large international conference dealing with all aspects of mercury pollution is arranged every second year^3^. Remote sensing of atomic mercury has been accomplished using the differential absorption lidar (light detection and ranging) method over ranges up to 1 km, related to industrial emissions (see, e.g.^4,5^) and to geothermal resources and mines^1,6,7^. There is a potential to discover valuable mineral resources by mercury vapor detection, since mercury is frequently coexisting with certain ores (see, e.g.^8^). Likewise, underground thermal resources might be revealed by surface mercury anomalies^9^. It could even be speculated on the use of localized atmospheric mercury anomalies as precursors to imminent seismic and/or volcanic activities. All these applications are challenging, but the demonstrated capability of extremely sensitive, range-resolved remote sensing of mercury gas is encouraging. In our quest to develop such geophysical exploration tools, we have pursued a much related project – the search for mercury escaping from the emperor Qin Shi Huang’s underground mausoleum in Xi’an – arguably the World’s most famous archeological site.

Emperor Qin, who unified China and ruled over the vast territory in 221–210 BC, has kept the fascination and interest of people, in particular after the 1974 discovery of the Terracotta Army protecting his mausoleum. The tomb is located in Lintong, 30 km north-east of present-day Xi’an and was constructed during 38 years from 246 BC, when he, at an age of 13, became king of a smaller region, up till 208 BC, two years after his death, which probably was caused by mercury poisoning. According to historian Sima Qian, who documented the life of the emperor^10^, the mausoleum and surrounding structures were built by 700 000 workers. He also stated, that the tomb chamber contained considerable amounts of liquid mercury, presumably forming rivers and lakes on a large-scale map of China. Further, mercury was at that time considered as an elixir of life. Mercury could be processed out of cinnabar (HgS) ore since ancient times^11,12^_._ The base of the pyramid-like tomb mound is about 380 × 330 m^2^ and it has a present height of about 50 meters. According to geo radar and gravimeter investigations, the underground palace has a size of about 140 × 110 × 30 m^3^ while the central coffin chamber measures 80 × 50 × 15 m^3^, with its ceiling about 30 meters below the present surrounding ground level^13,14^. Current understanding is that the chamber has never been opened/looted, and a detailed archeological investigation is postponed awaiting improved methods to safeguard artifacts from air exposure and swift degradation.

Our hypothesis in the current study was that, if mercury, at the amounts as estimated at 100 tons or more, would have been introduced into the tomb chamber, then the likely formation of cracks in combination with the high natural vapor pressure of mercury would result in a faint still ongoing emission of atomic mercury vapor from the mound, and should be detectable by mapping the surrounding air. Actually, mercury, unlike other elements, stays in atomic form in the atmosphere, and the differential absorption lidar (DIAL) technique^15^ is able to measure atmospheric mercury concentrations down to the Atlantic background level of about 1–2 ng/m^3^ if averaged over a 1-km range^1^. In ancient China, it seems that it became a custom to introduce mercury into the tombs of the nobility. In the Song Dynasty (960–1279), after the death of high-ranked persons like a minister, mercury was given for graves as a reward from the emperor^16,17^. Mercury fumes were in fact used in the localizing of a tomb in the Chinese city of Hancheng^18^. Actually, our preparations for the present measurements were initiated already in 2009, but could not for different reasons, including legislation, be performed until now.

Systematic measurements of the soil covering the Qin mausoleum mound have actually revealed elevated levels of mercury, up to 1440 ppb and with an average value of 205 ppb in one study, which is reported in ref. ^19,20^, while a newer investigation found 2204 ppb and 169 ppb, respectively^21^, considerably higher than typical background values for the area. Studies of mercury related to the Qin mausoleum are discussed in^22^, while corresponding mercury studies in the open atmosphere have to our knowledge not been performed earlier. Clearly, any measurements of atmospheric mercury of possibly archaeological origin have to carefully consider mercury levels due to environmental pollution, which is high in the Xi’an area, and wind direction monitoring is clearly important.

**Figure 1:**
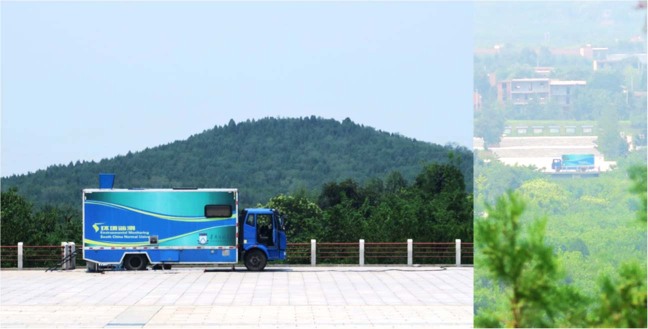
Measurement site situation. The SCNU mobile differential absorption lidar system on site #1 (as indicated in Fig. 2) in front of the Qin mausoleum, which is seen in the background. A view towards the lidar system as seen from the top of the pyramid is shown to the right.

**Figure 2:**
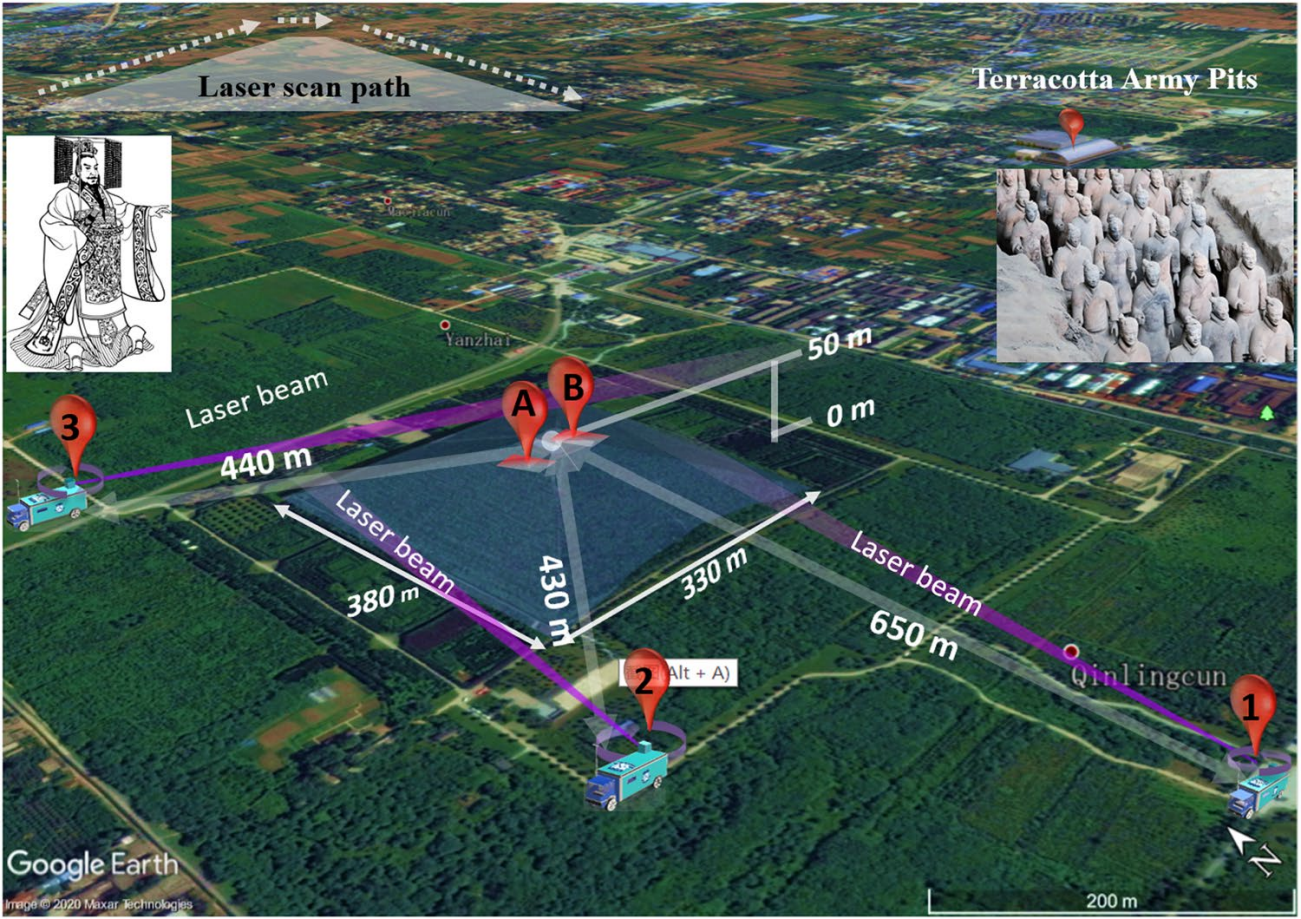
Schematic view of the measurement site with the Emperor Qin Mausoleum and the Terracotta Warrior site. The three measurement positions (#1, #2, and #3) of the lidar system are shown with distances indicated. Inserts show a photograph of part of the about 8000 man strong army, as well as an ancient drawing of Emperor Qin. A and B indicate the locations of highest soil mercury content according to^19,20^ and^21^. (Satellite imagery from Google Earth; photograph by S. Svanberg; Emperor Qin picture from Wikimedia Commons Qin_Shi_Huang_BW.png).

During the time period July 24 to August 12, 2016, with very hot weather favoring emission (typical daytime temperatures reaching 35 degrees and night-time temperatures around 30 degrees) we performed extensive measurements of the atmospheric mercury content around the tomb. We used a mobile differential absorption lidar system^23^, recently constructed, largely for this particular purpose, by the South China Normal University Applied Laser Spectroscopy Group. Lidar is a remote-sensing technique able to measure atmospheric gases by transmitting a pulsed laser beam and observing the back-scattered light from different range intervals. The range-resolution achievable depends on the concentration of the gas, and is degrading with lower concentrations. The system is shown in Fig. 1 in one of the measurement positions used (#1), about 650 meters from the top of the tomb mound. A photograph of the system taken from the top of the pyramid is also included. Figure 2 gives an overview of the measurement area also showing the location of the Terracotta army pits about 2 km to the east of the tomb pyramid. Our three different measurement sites surrounding the pyramid are indicated in the figure by 1, 2, and 3. Further, the locations with highest soil mercury content according to^19,20^ and^21^ are indicated on the mound by symbols A and B, respectively. A weather station was installed at the top of the tomb mound, and a further one at the lidar system, at each location about 7 meters above the surrounding ground level.

## Measurement System

The lidar system, which is described in detail in^23^, is, depending on atmospheric conditions, capable to map the concentration of atomic mercury out to a range of about 1 km, and can have a concentration sensitivity approaching typical Atlantic background levels of mercury, thus putting us in a good position to observe any anomalies. Generally speaking, the conditions for reaching a high sensitivity in absorption spectroscopic measurements are explained by the common Beer-Lambert law, which states that a high transition probability, such as for mercury, is always favourable, and that a low concentration can be compensated by a long distance over which the absorption is evaluated. Elevated concentrations, such as those found in our experiments, are needed to allow a reasonable range resolution, taking advantage of the differential absorption lidar concept. The site under study, being close to a major Chinese city, was found to have a general elevated mercury pollution level, on top of which hotspots related to the tomb were to be identified. The mercury concentrations measured allowed the use of an effective range resolution of 30 meters.

Our system, built with inspiration from a Swedish mobile system^24^, is based on a Jiefang truck, carrying a 7.5  × 2.4 × 2.4 m^3^ laboratory container. Narrow-band pulsed laser radiation at the mercury absorption wavelength close to 254 nm, was generated by a frequency-doubled dye laser, pumped by 355 nm radiation from a Nd:YAG laser operating at 20 Hz repetition frequency. The energy of the UV laser pulses used for pumping was up to 650 mJ, and with the dye Coumarin 307 dissolved in ethanol, green pulses at 508 nm were generated, which after frequency doubling yielded up to 15 mJ in 10 ns pulses at the relevant mercury wavelengths and with a linewidth better than 0.13 cm^−1^.

The wavelength was switched, alternating between a wavelength (λ_on_) at 253.729 nm, where mercury has maximum absorption and a nearby, non-absorbed reference wavelength (λ_off_) at 253.744 nm while avoiding interference from near-by molecular oxygen lines. Detailed spectroscopic considerations regarding the overlapping structure of hyperfine and isotope-shift components, all pressure broadened into a quite smooth atmospheric pressure line, as well as regarding the molecular oxygen close-lying lines, can be found in ref. ^25^ The two wavelengths are sufficiently close so that a single frequency-doubling crystal at a fixed phase-matching angle could efficiently be used for both wavelengths. The frequency of every laser pulse was measured with a precision wavelength meter (Highfinesse WS-6), the output of which was fed back to a piezo actuator in the dye laser, in this way stabilizing the laser frequency in a closed-loop circuit. The linewidth and wavelength stability were ascertained by measurements on narrow oxygen absorption lines as detailed in ref. ^23,25^.

Back-scattered light is collected by a vertically looking 40 cm Newtonian telescope, over which a computerized folding mirror is placed to allow spatial scans. Signals for the on- and off-wavelengths are averaged in separate memories, and finally, the signals are divided resulting in a ratio curve. A slope of this curve reveals a mercury concentration^15^, with higher slopes corresponding to higher concentrations, which follows as a direct reflection of the well-known Beer-Lambert law. The differential absorption cross section 2.5*10^−14^ cm^2^/atom was employed in the spectroscopic evaluation of mercury concentrations^23^. It should be mentioned, that the validity of this process was assessed in an independent inter-calibration campaign of our remote-sensing system towards a conventional point monitor (Lumex RA-915M), used in the large global mercury research community, with satisfactory agreement as reported in ref. ^7^ The absorption cross section has a temperature and pressure dependence, but since the temperature influences only with a square root dependence, and since the elevation of Xi’an is only 400 m, the correction to actual conditions is negligible compared to other error sources. Also, considering that the wavelength separation between on two wavelengths used in the DIAL measurements is very small, the lidar curve division to obtain the DIAL curve becomes free of errors due to any differential effects in the particle backscattering.

## Measurements and Results

**Figure 3:**
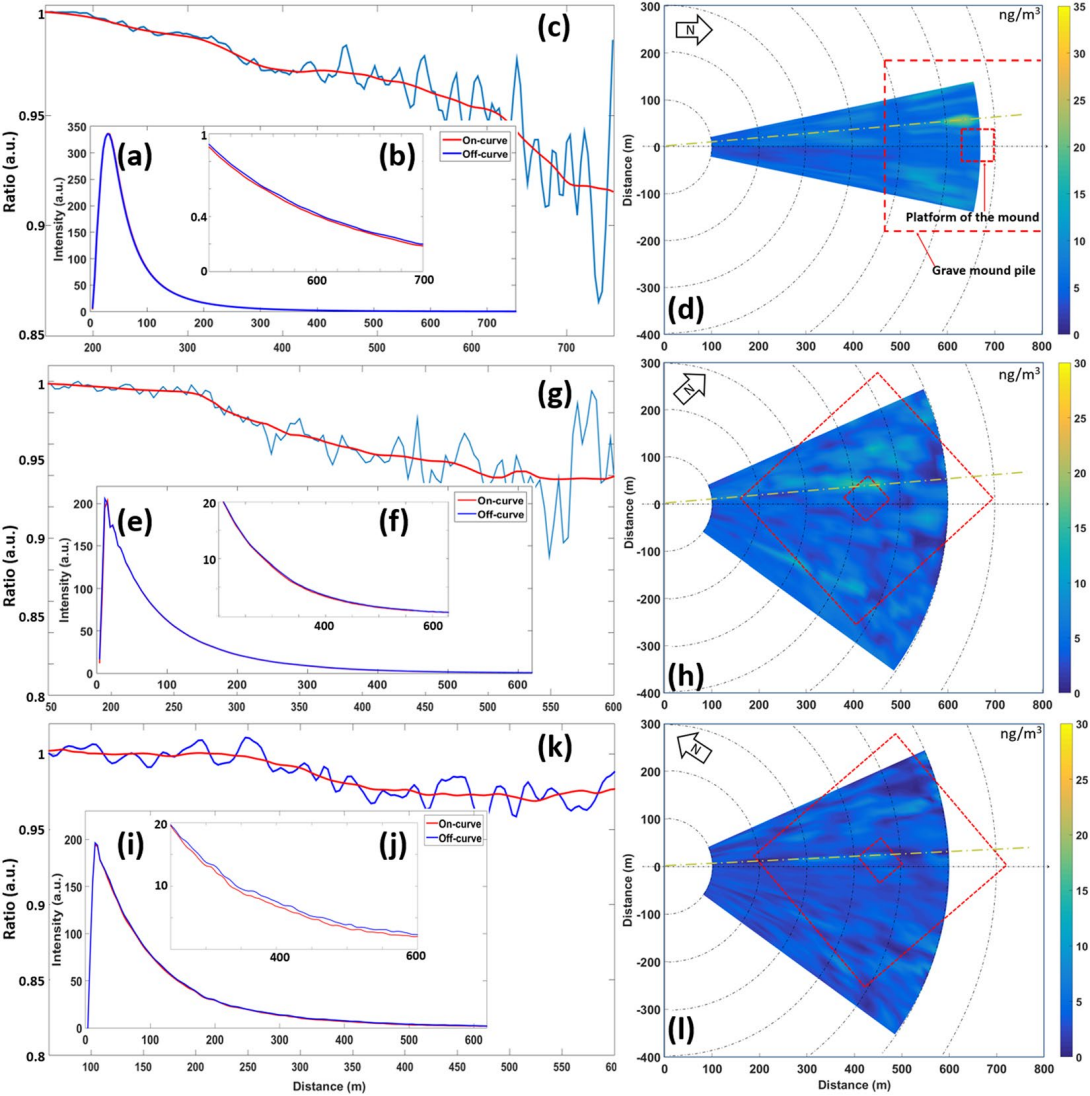
Data for atomic mercury monitoring at the Emperor Qin mausoleum. Panels (**a**–**d**) correspond to measurement location #1, (**e**–**h**) to location #2, and (**i**–**l**) to location #3. Curves (**a**,**e**,**i**) are recorded lidar backscattering signals, (**b**,**f**,**j**) are magnified sections of the curves, where recordings for on- and off-absorption wavelengths close to 254 nm are shown individually in red and blue, respectively. DIAL curves, obtained by dividing on- by off-resonance recordings are finally shown in (**c**,**g**,**k**). These curves would have a constant value of 1.00 (apart from noise) for all ranges in the absence of atmospheric atomic mercury, but are in fact sloping, where a larger slope corresponds to a higher gas concentration. Mercury concentration maps over the mound are shown in panels (d,h,l), as obtained from locations #1, #2, and #3, with the concentrations given in ng/m^3^ and color-coded, to be evaluated against the color scales to the right of the maps. Here also the measurement directions corresponding to the individual data shown to the right of each map are indicated by yellow dashed lines. Since the maps are generated by averaging over several DIAL curves of the type shown in **(c,g,k)**, the colored map data along the yellow lines are not fully matching the individual raw data curves shown.

In searches for mercury concentration anomalies around the mausoleum, the laser beam was scanned above the profile of the mound as indicated in the inset in the upper left part of Fig. 2, with the beam at a closest distance of about 5 m from the mound, which is mostly planted with pomegranate trees. An example of recorded data obtained from measurement point #1 with the laser beam directed just above the top of the mound is shown in Fig. 3(a–c). Figure 3(a) is a direct recording of the lidar backscatter signal, while (b) is a magnification of the signal from the range interval 500–700 m. The DIAL signal ratio curve is shown in Fig. 3(c), where a clear slope is shown, with a particularly strong feature at a range of about 650 m, indicating elevated mercury concentrations. The data were obtained by averaging 540 pairs of on/off wavelength pulses during a time of about 1 minute. At far range, the curve becomes noisier, reflecting the fact that the backscattered intensity falls off with an inverse range-squared dependence. The raw DIAL curve is shown as a blue line. The red line, used for the concentration evaluation, is obtained by smoothing the DIAL curve in applying a sliding averaging over a 30 m range. Most of our measurements were performed at nighttime, when ambient light level is low. In this way the background in our optical recordings was reduced and signal-to-noise levels were improved. In principle, at 254 nm, no ambient radiation is present due to the absorption of stratospheric ozone with a cut-off at 300 nm, but some daylight leaked through the interference filter used in front of the detector. Since ozone absorption is unstructured, no influence on our mercury measurements from possible tropospheric ozone is experienced. The attainable measurement range was limited to about 700 m, since the short-wavelength light is strongly attenuated by scattering in the misty atmosphere connected to high temperature and general levels of particulate pollution. Longer ranges as indicated in the introduction, are achievable in clear atmosphere conditions, allowing better laser beam penetration. Data evaluation is normally initiated at a range of 100 m, since for closer ranges small laser beam overlap deviations as well as possible saturation of the atomic transition can degrade the data accuracy.

Evaluated averaged concentrations based on recordings of the type shown in Fig. 3(c) from full scans over the mound are shown in Fig. 3(d), where the measurement direction corresponding to Fig. 3(c) is specially indicated by a yellow dashed line. The curve exhibits a weak over-all slope indicating a background concentration of about 5–10 ng/m^3^ and an elevated concentration of about 23 ng/m^3^ on the western slope, close to the mound top platform. Elevated concentrations are also noted on the eastern slope, slightly towards the south. A full scan, comprising about 60–80 individual measurement directions, took about 4 hours, including the time for data transfer, mechanical movement of the scanning dome between the measurement directions, and spatially adjusting the somewhat complex scan, with an upward slope, a flat top section followed by a downward slope. The data from three such scans were then processed into the concentration map shown in Fig. 3(d). The average wind speed and direction during the data recording, as evaluated from the weather station on the top of the mound, were 2.3 m/s and coming in from the East-North-East.

Data recorded from measurement position #2 as indicated in Fig. 2 are shown in Fig. 3(e–h). The concentration map obtained from this position as averaged over two complete scans over the mound is shown in Fig. 3(h). We again note an area with elevated concentrations on the western slope, now raising to about 22 ng/m^3^ in a typical surrounding level of about 5–10 ng/m^3^. A further area with elevated concentration is noted close to the southern base. Average wind data during the recording were 2.7 m/s from the East-North-East.

Finally, data from measurement site #3 are shown in Fig. 3(i–l). Again, the concentration map was the result of two full scan. No evident concentration hotspots were observed in this case, with highest concentrations reaching 10 ng/m^3^.The average concentration over the total area of the mound was 6 ng/m^3^. During the recording of the data from site #3, the average wind data were 5.4 m/s, again from the East-North-East. This is higher by a factor of two than for the maps shown in Fig. 3(d,h) (the wind speeds there were 2.3 and 2.7 m/s, respectively), and leads to a corresponding strong dilution of a localized mercury emission, which fully explains the absence of hotspots in this case. When the wind speed increases by a factor of two, the concentration due to a localized source is reduced by a factor of two, since the emission flux is the same. Flux issues are further discussed below.

We note, that the generation of such concentration maps is not trivial, mainly since the atmosphere is changing and the concentrations are varying, requiring a substantial amount of range smoothing, through which spatial resolution is reduced. Thus, while increased and localized concentrations were observed at certain locations over the tomb, as exemplified in Fig. 3(d,h), the concentration values, averaged over time and area to lower values, they are still significantly elevated compared to the surrounding area, and especially high in the areas previously indicated as hot-spots^19–21^. Occasionally quite high concentrations were observed in individual recordings, such as shown in Fig. 3(c). The DIAL curve slope observed at 650 m distance, right over one of the suggested hotspots, corresponds to a concentration value of 27 ng/m^3^. Occasionally, during the measurement campaign, even higher concentrations were observed. We argue, that a high localized value (23 ng/m^3^) of atmospheric mercury, persistent in three averaging scans as shown in Fig. 3(d), can only be explained by a localized emission from the mound. This conclusion is reached, when considering all the aspects influencing the accuracy of the measurements, which at the measurement range pertaining to the hot-spots in Fig. 3(d,h) is estimated to be ± 5 ng/m^3^. The accuracy, which for a chosen evaluation range interval is strongly dependent on the signal-to-noise ratio in the DIAL curve data, is clearly considerably higher for shorter ranges in the mercury concentrations maps given in Fig. 3(d,h,l). As mentioned above, the general background concentration levels in the area were about 5–10 ng/m^3^ with a highest observed value, apart from the Qin mausoleum location, of 13 ng/m^3^, possibly related to a further archaeological feature (unpublished data), which should be further investigated.

The question if possible mercury pollution from the Qin tomb significantly influences the surrounding environment has been addressed by Jin *et al*.^21^, who performed measurement of mercury concentration in soils and water. The lidar technique can in principle measure the total flux of a gaseous pollutant out from a source or an area, by performing vertical scans downwind of the source. This has been effectively demonstrated for strong mercury emission sources such as chlor-alkali plants, which employ liquid mercury electrodes^5^. Then, the concentration as integrated over the full cross-section area of the emission plume, as defined by the laser scan, is first determined. The local concentration, with unit [kg/m^3^], is added up horizontally (unit [m]) and vertically (unit [m]) yielding the area-integrated value, with unit [kg/m^3^ x m x m] = [kg/m]. Finally, multiplying this value with the perpendicular wind velocity, with unit [m/s]), we can obtain the total flux, which has the expected unit [kg/s].

To estimate the total net flux of atmospheric mercury from the mausoleum is, however, much more challenging, since the concentrations are much lower, the ambient air already contains elevated and varying concentrations of mercury, and the wind field around the mound is complex and varying. Such vertical scan measurements were tried and revealed maximum concentrations up to 8 ng/m^3^ in the presence of wind speeds of 5 m/s (unpublished data). Flow from the detected hot spots led to lower maximum concentrations as diluted by the comparatively high wind speed (similar to the case of the mound measurements at site 3) and turbulence downwind from the mound. Also, because of the rather high general background concentration levels, an integrated downwind flux value would need to be subtracted by an integrated upwind, likewise very uncertain, flux value. Thus, our efforts did not lead to any reliable measurements of the net total outflux from the tomb. Instead, an extremely crude estimate of the flux was made as presented below.

## Conclusions

Differential absorption lidar mapping of the atmospheric mercury concentrations around the mausoleum of the first Qin emperor was performed, as we believe, for the first time. Measurement ranges were up to 700 meters. Elevated concentrations of mercury were noted around the mausoleum mound, with values raising to around 25 ng/m^3^ compared to ambient concentrations of typically 5–10 ng/m^3^. The estimated accuracy of our concentration measurements is about ± 5 ng/m^3^ at the range of the hot-spots, and better for closer ranges.

Areas of highest concentrations on the pyramid slopes show correlation with earlier findings of elevated soil mercury concentrations^19–21^. Based on the data presented in Fig. 3(d,h) with the experimentally determined size of the area of increased mercury concentration (about 10 ng/m^3^ excess concentration, above the ambient concentrations), the wind speed and direction, and very crudely assuming the mercury plume to uniformly extend up to 20 m above the surface of the mound, we estimate a flux of mercury out of the pyramid of about 5 *10^−8^ kg/s. Clearly, this number has a large uncertainly, maybe of a factor 2, but can be used as a starting point for a calculation example for speculations on possible amounts of deposited mercury in the tomb. If we make the extremely uncertain assumption of a constant rate of emission to air (at the amount inferred from our measurements) during the 6 warmest months of the year and during half the time span since the closure of the tomb, we obtain a total loss of mercury to air of the order of 1 ton of liquid mercury. Clearly, there are very large uncertainties in these estimations, but our findings add to the credibility of 2200 year-old records by historian Sima on the existence of large amounts of mercury in the Emperor Qin tomb^10^, also in view of estimates of the production capability of mercury at the times of Emperor Qin^11,12^.

We note that the surface soil content of mercury has earlier been used as a similar evidence. If we assume that the measured average concentrations of deposited mercury in the soil (190 ppb^19–21^) to be representative for the earth masses in the whole mound, we would likewise get a number of about 1 ton of mercury. It has speculatively been suggested that the actual pattern of surface mercury on the mound would reflect the mercury distribution on the underground palace map of China. However, since the mercury vapor distribution in the underground palace would be uniform, irrespective of the detailed localizations of liquid mercury, such claims would be unwarranted. Our present measurements on gaseous mercury further show, that the Emperor Qin mausoleum does not contribute in a significant way to the atmospheric mercury concentrations in the area, similarly as the water and soil mercury levels in the surrounding areas were found not to be significantly elevated^21^. The successful observation of weak mercury emission due to a deep-lying underground source also illustrates a potential for using mercury as a tracer gas for natural resource prospecting^1,8,9^, which is intended.

**Figure 4:**
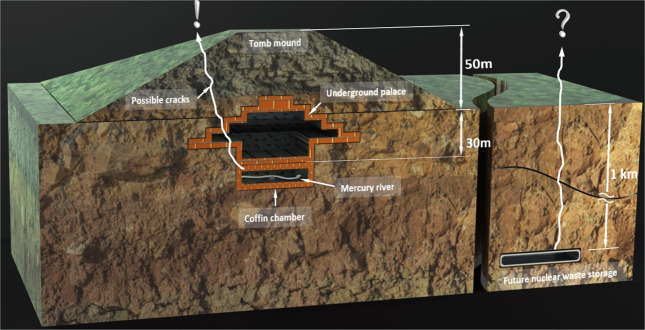
Emperor Qin’s tomb and future long-term storage of nuclear waste – the issue of constructing a “permanent” enclosure – an artist’s conception.

Our observations of leaks out of an underground chamber, intended to be intact and sealed “forever” for in- and out-flux, bring the current quests of constructing a suitable underground permanent deposit of nuclear waste to mind^26–29^. A US facility for weapon-related waste, WIPP (Waste Isolation Pilot Plant) is operating, but not without problems^30^. Sweden and Finland have facilities in construction (in Östhammar and Posiva, respectively), and further countries are in different project stages. Abundant fission products like ^90^Sr and ^137^Cs have medium half-lives of tens of years, while long-lived isotopes such as ^93^Zr and ^135^Cs have a half-life above one million years. Like for the ancient constructors at Xi’an, the task is now to ensure a secure enclosure, unaffected by possible geophysical events. An artist’s conception of the two scenarios is presented in Fig. 4. Leaking radioactive compounds would be detectable using the radioactivity itself, including the associated radon gas. So far, the nuclear waste is largely stored on the power plant sites waiting for the construction of suitable final deposit sites. Our study shows that leakage of dangerous material from an underground storage site can be a reality in a time span of 2000 years, even if great precautions were taken to seal the tomb. Regarding final storage of radioactive material, there is a widespread public doubt – possibly unwarranted - that present day engineering could achieve a completely safe solution, which is hampering constructions, leaving the general long-term nuclear waste storage a still somewhat open issue. Different time epochs have their own geophysical challenges to handle.
